# Exploring gene biomarkers and targeted drugs for ferroptosis and cuproptosis in osteosarcoma: A bioinformatic approach

**DOI:** 10.1002/tox.24250

**Published:** 2024-03-28

**Authors:** Yingnan Ji, Lv Liu, Yu Liu, Yudong Ma, Zhenhua Ji, Xiaodan Wu, Qi Zhu

**Affiliations:** ^1^ Central Hospital Affiliated to Shenyang Medical College Shenyang China; ^2^ Benxi Central Hospital Benxi China

**Keywords:** cuproptosis, ferroptosis, osteosarcoma, prognostic marker

## Abstract

Osteosarcoma predominantly affects adolescents and young adults and is characterized as a malignant bone tumor. In recent decades, substantial advancements have been achieved in both diagnosing and treating osteosarcoma. Resulting in enhanced survival rates. Despite these advancements, the intricate relationship between ferroptosis and cuproptosis genes in osteosarcoma remains inadequately understood. Leveraging TARGET and GEO datasets, we conducted Cox regression analysis to select prognostic genes from a cohort of 71 candidates. Subsequently, a novel prognostic model was engineered using the LASSO algorithm. Kaplan–Meier analysis demonstrated that patients stratified as low risk had a substantially better prognosis compared with their high‐risk counterparts. The model's validity was corroborated by the area under the receiver operating characteristic (ROC) curve. Additionally, we ascertained independent prognostic indicators, including clinical presentation, metastatic status, and risk scores, and crafted a clinical scoring system via nomograms. The tumor immune microenvironment was appraised through ESTIMATE, CIBERSORT, and single‐sample gene set enrichment analysis. Gene expression within the model was authenticated through PCR validation. The prognostic model, refined by Cox regression and the LASSO algorithm, comprised two risk genes. Kaplan–Meier curves confirmed a significantly improved prognosis for the low‐risk group in contrast to those identified as high‐risk. For the training set, the ROC area under the curve (AUC) values stood at 0.636, 0.695, and 0.729 for the 1‐, 3‐, and 5‐year checkpoints, respectively. Although validation set AUCs were 0.738, 0.668, and 0.596, respectively. Immune microenvironmental analysis indicated potential immune deficiencies in high‐risk patients. Additionally, sensitivity to three small molecule drugs was investigated in the high‐risk cohort, informing potential immunotherapeutic strategies for osteosarcoma. PCR analysis showed increased mRNA levels of the genes FDX1 and SQLE in osteosarcoma tissues. This study elucidates the interaction of ferroptosis and cuproptosis genes in osteosarcoma and paves the way for more targeted immunotherapy.

## 1 INTRODUCTION

Osteosarcoma, the foremost primary malignant tumor of bone, is characterized by a propensity for local invasion and metastasis. Despite significant progress in surgical and chemotherapeutic treatments, the outlook for patients with metastatic or recurrent osteosarcoma is still poor. Immunotherapy has become a leading approach in combatting various malignancies, illuminating the role of the immune system in osteosarcoma and enhancing immunotherapy's effectiveness through the identification of biomarkers, thus broadening its applicability. It incorporates immune modulators, checkpoint inhibitors, and combination therapies, significantly reducing treatment‐related side effects, improving efficacy, and improving quality of life.^1^ The field is now increasingly focused on identifying novel biological targets for future therapies. Ferroptosis, recently identified as a distinct form of cell death, plays a crucial role in a multitude of physiological and pathological processes, including cancers, with major regulators such as GPX4 and Nrf2 being crucial in tumor dynamics.^2^ The Nrf2/xCT/GPX4 axis is known to counteract osteosarcoma.^3^ In 2022, Todd R. Golub's team introduced cuproptosis, a unique programmed cell death pathway distinct from classical apoptotic and necrotic pathways and driven by protein–lipid interactions in mitochondria due to copper toxicity. This toxicity disrupts the TCA cycle, resulting in detrimental protein aggregations, particularly affecting the pyruvate dehydrogenase complex, and ultimately leading to cell death.^4^, ^5^, ^6^, ^7^, ^8^, ^9^ Research shows that osteosarcoma tumors require extensive energy, likely derived from glycolysis and the TCA cycle.^10^ This study delves deeper into the role of copper in mitochondrial stability and its broader implications in cell biology,^11^, ^12^ providing new angles for using copper‐mediated mechanisms in cancer treatment. By using comprehensive tumor tissue sequencing data, we have developed an osteosarcoma prognosis model based on the interaction between ferroptosis and cuproptosis pathways. This model is poised to enhance clinical prognosis and aid in molecular drug development.^13^, ^14^ Our study extends beyond existing models such as those based on apoptosis and RNA methylation‐related genes by combining genes implicated in both ferroptosis and cuproptosis, creating an innovative prognostic tool with the potential to improve clinical decisions. The model also evaluates the relationship between prognosis and immune response, potentially leading to more effective targeted therapies for osteosarcoma.^15^, ^16^ In summary, forging an effective osteosarcoma prognostic model constitutes a vital advance in the amelioration of diagnosis, prognosis, and therapy for this aggressive cancer.

## 2 MATERIALS AND METHODS

### 2.1 Collection of osteosarcoma datasets

The osteosarcoma dataset combines data from two principal sources: the TARGET and the GEO database. The TARGET database contains 88 tumor samples, GSE21257 provides 53 samples, and GSE16102 includes 57 samples following the removal of three human embryonic cell lines and six normal tissue samples. To maintain uniformity, the data from various platforms underwent normalization. Pertinent literature has identified 60 genes connected to ferroptosis and 11 genes related to cuproptosis.^17^, ^18^ The TARGET database serves as the training set, with GSE21257 and GSE16102 acting as validation sets.

### 2.2 Expression patterns and interactions of co‐genes

To characterize the expression patterns and interactions among co‐expressed genes, we employed a suite of graphing packages such as “igraph,” “psych,” “reshape2,” and “RcolorBrewer.” Selection of these packages was based on their proven capacity for visually rendering complex data and ensuring its coherent representation.

### 2.3 Construction of the signature using the LASSO regression model

We performed a thorough analysis of 71 genes implicated in ferroptosis and cuproptosis using univariate Cox regression to identify those with a *p*‐value below .001 for inclusion in a LASSO regression model. The model stratified the samples into high‐risk and low‐risk categories based on their respective risk coefficients. Survival curves were then generated for each category using the “survival” package, with the model's precision evaluated by receiver operating characteristic (ROC) curves derived from the ‘survivalROC’ package. Additionally, expression profiles of the prognostic genes in both risk categories were validated via univariate and multivariate Cox regression analyses, enabling the identification of independent prognostic risk factors. Our findings shed light on the significance of these genes in ferroptosis and cuproptosis and their utility as prognostic biomarkers.

### 2.4 Nomogram prognostic model construction

We developed a prognostic nomogram utilizing clinical indicators deemed significant by a multivariate Cox regression analysis, with a *p*‐value of .001 or less indicative of statistical significance. The model's performance was assessed using calibration curves and the concordance index (c‐index). To confirm the model's predictive accuracy, we constructed ROC curves for 1‐, 3‐, and 5‐year intervals. Additionally, Kaplan–Meier survival analysis was conducted on high‐risk groups, confirming statistical significance with a *p*‐value less than .05.

### 2.5 Cell infiltration in the tumor microenvironment

Utilizing gene set enrichment analysis (GSEA), we compared two risk groups to delineate potential biological functions associated with our risk scoring framework, employing the c2.cp.kegg.v7.0.symbols.gmt gene set with 1000 permutations for intergroup comparisons and without the need for gene name conversion. Utilizing the single‐sample gene set enrichment analysis (ssGSEA) algorithm, we quantified the presence of infiltrating immune cells within the tumor microenvironment for each sample. To assess the variance in immune cell infiltration between the high‐risk and low‐risk cohorts, the CIBERSORT algorithm was employed. We also leveraged the “estimate” package to investigate the relationship between the risk score and multiple factors, such as immune score, ESTIMATE score, stromal score, and tumor purity, and thereby evaluate their connection with prognostic indicators. Additionally, we deployed the tumor immune dysfunction and exclusion (TIDE) algorithm with the objective of predicting responses to immune checkpoint inhibitors in individual samples or subtypes, as delineated in previous research.^19^

### 2.6 Small molecule drug screening based on risk model

The IC50 values are quantified using the ‘pRRophic’ package in R. Drugs with potential relevance to osteosarcoma are analyzed based on the median‐risk score.

### 2.7 Cell culture and gene expression

The human osteoblast cell line hFOB 1.19 and the human osteosarcoma cell line MG63 were obtained from the CAS cell bank. The hFOB 1.19 cells were propagated in DMEM/F‐12 complete medium (The number is KGM41500N‐500), whereas MG63 cells were grown in α‐MEM complete medium (The number is KGM44892N‐500), with both media supplemented with 10% fetal bovine serum (The number is KGY008, Sangon Biotech, Inc. Shanghai China, FBS). Cultures were maintained in a controlled environment at 37°C and 5% CO2. Total RNA was extracted from the cells and osteosarcoma tissue samples using TRIzol reagent (The number is RR047A, Invitrogen, Carlsbad, CA, USA), in accordance with the manufacturer's instructions.

Subsequent to RNA isolation, to synthesize cDNA, one microgram of total RNA underwent reverse transcription using the RevertAid First Strand cDNA Synthesis Kit (Thermo Fisher Scientific). Subsequently, quantitative real‐time PCR (qRT‐PCR) was conducted utilizing SYBR Green Mix (Vazyme, China). The amplification protocol included an initial denaturation at 95°C for 1 min followed by 35 cycles of denaturation at 95°C for 90 s, annealing at 60°C for 30 s, and extension at 72°C for 30 ss, with a final extension step at 72°C for 10 min. Relative gene expression levels were quantified using the 2^‐ΔΔCt method, with GAPDH serving as a reference gene. The primer sequences used for qRT‐PCR are as follows: GAPDH forward 5′‐CTGAGTACGTCGTGGAGTCC‐3′ and reverse 5′‐GTCTTCTGGGTGGCAGTGAT‐3′; SQLE forward 5′‐GGCCTGCCTTTCATTGGCTT‐3′ and reverse 5′‐TTCCTTTTCTGCGCCTCCTG‐3′; FDX1 forward 5′‐TCTGCTGTCCTCGGCGG‐3′ and reverse 5′‐GGTTCCCTCACATGCACCAAA‐3′ (The primer sequence designed by Beijing Bioss, China).

### 2.8 Statistical analysis

All statistical analyses were conducted using R version 3.6.1. Experimental results are presented as mean values with their respective standard deviations. Statistical significance was established using one‐way ANOVA. Survival differences between groups were compared using Kaplan–Meier curves and log‐rank tests, with significance set at a two‐sided *p*‐value of less than .05. Each experiment was performed a minimum of three times.

## 3 RESULTS

### 3.1 Expression patterns of ferroptosis and cuproptosis associated genes in osteosarcoma

**FIGURE 1:**
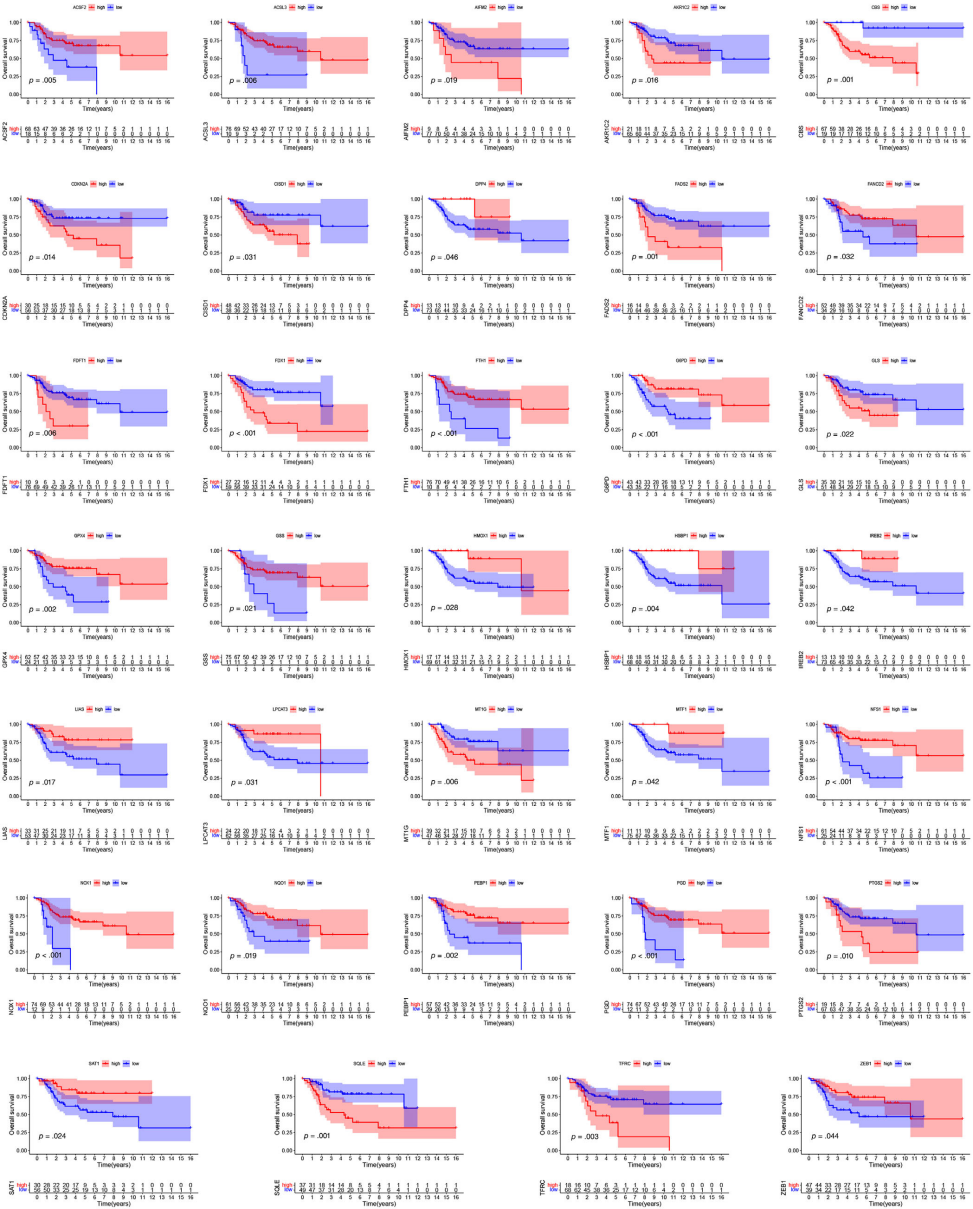
Kaplan–Meier curves for the 34 genes in osteosarcoma patients from TARGET database.

**FIGURE 2:**
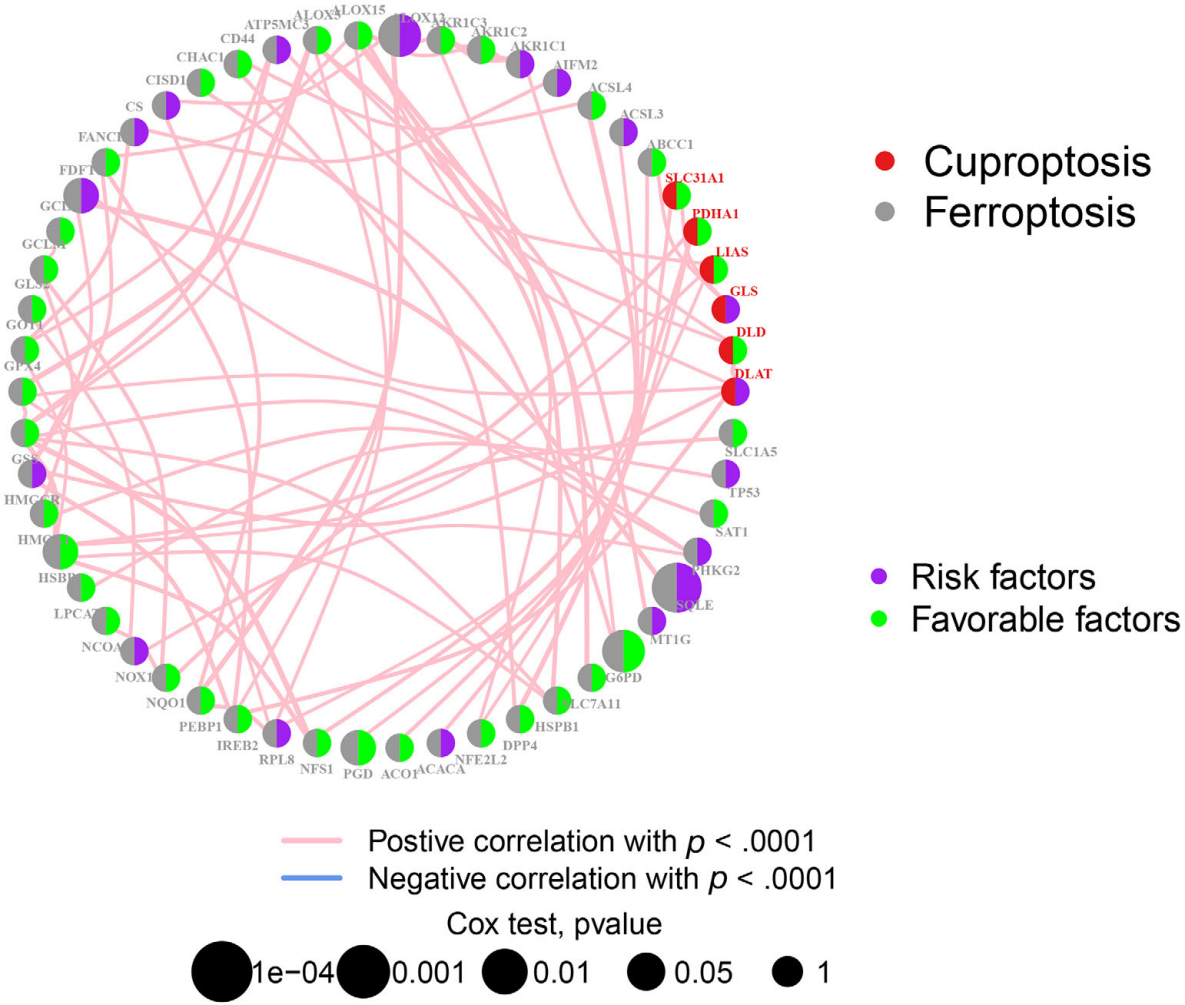
A network of correlations including FRGs and CUGs in the TARGET cohort. (*p < .05; **p < .01; ***p < .001).

To elucidate the prognostic significance of ferroptosis and copper‐induced cell death‐related genes in osteosarcoma, we conducted a comprehensive analysis of 71 pertinent genes and their correlation with patient survival outcomes (refer to Figure 1). Moreover, we have constructed a network diagram (refer to Figure 2) that delineates the interactions and potential prognostic relevance of these genes. The analysis uncovered that 34 regulator genes demonstrated a significant association with survival outcomes. Noteworthily, an elevated expression of certain genes, including ACSF2, ACSL3, DPP4, FANCD2, FTH1, G6PD, GPX4, GSS, HMOX1, HSBP1, IREB2, LIAS, LPCAT3, MTF1, NFS1, NOX1, NQO1, PEBP1, PGD, SAT1, and ZEB1, was linked to improved prognosis. Conversely, lower expression levels of AIFM2, AKR1C2, CBS, CDKN2A, CISD1, FADS2, FDFT1, FDX1, GLS, MT1G, PTGS2, SQLE, and TFRC were associated with a more favorable prognosis compared to their higher counterparts.

### 3.2 Construction of a prognostic model based on co‐expressed genes

**FIGURE 3:**
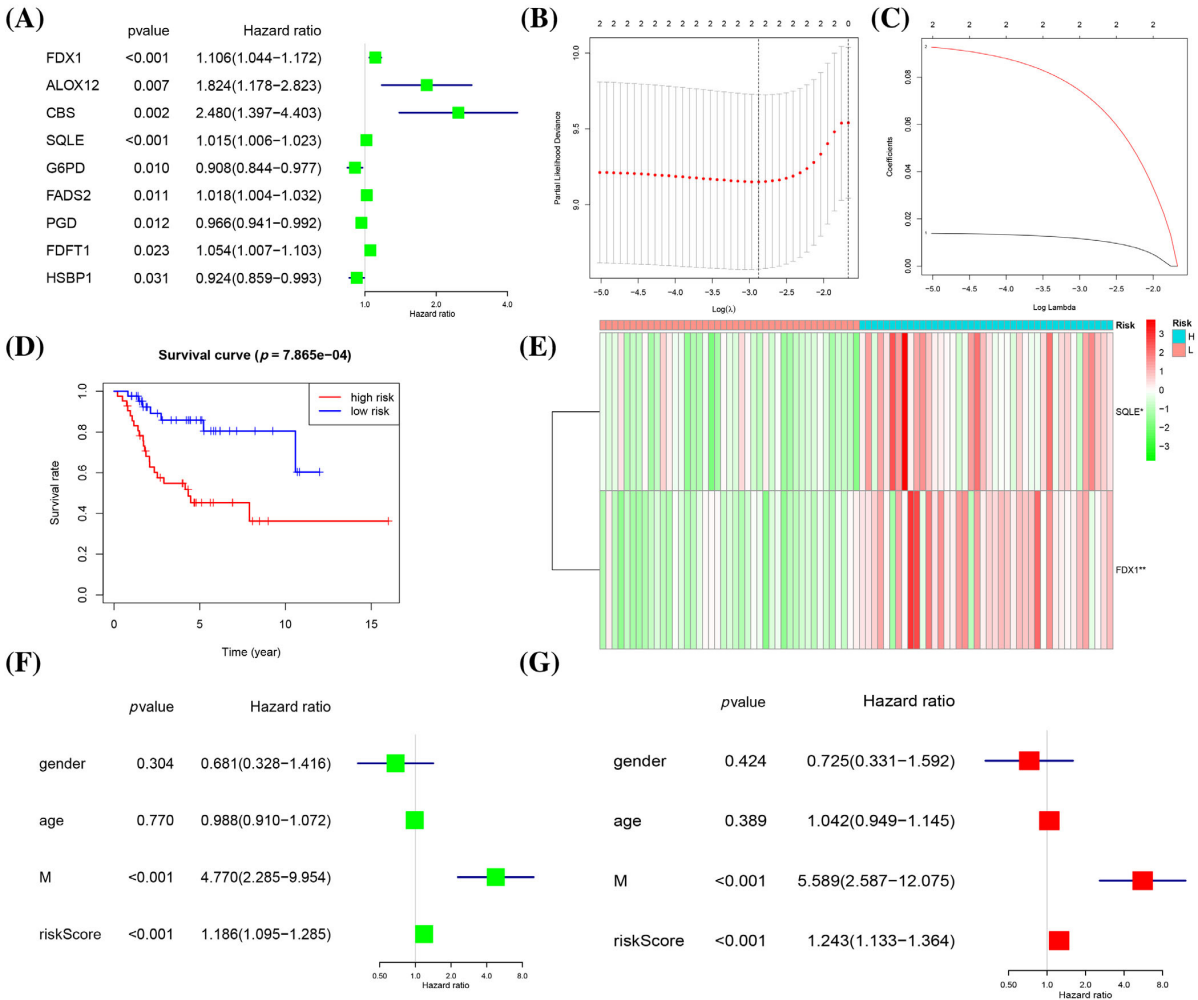
Prognostic relevance and construction of the risk signature of FRGs and CUGs in osteosarcoma (OS). (A) The prognostic analyses for nine genes using univariate Cox regression model. (B, C) LASSO coefficient profiles of the two genes. (D) The Kaplan–Meier analysis showed that patients in the low‐risk group presented better osteosarcoma than those in the high‐risk group for training set. (E) Expression patterns of two selected prognostic genes in high‐and low‐risk groups for training set. (F, G) The training set of forrest plot of the independent prognostic factors in OS.

A univariate Cox regression analysis was conducted on 71 genes, resulting in the identification of nine significant genes: FDX1 (*p* = .000597), ALOX12 (*p* = .007014), CBS (*p* = .00191423), SQLE (*p* = .00041), G6PD (*p* = .00968), FADS2 (*p* = .0112), PGD (*p* = .01182), FDFT1 (*p* = .0235), and HSBP1 (*p* = .031) (Figure 3A). Remarkably, both FDX1 and SQLE demonstrated profound significance (*p* < .001) and were consequently incorporated into the LASSO regression algorithm to construct a two‐gene prognostic risk model (Figure 3B,C). Using the median risk score as a cutoff, subjects were dichotomized into high‐risk and low‐risk groups. Noteworthy is the significantly enhanced survival observed in the low‐risk group compared with the high‐risk group (Figure 3D). In the training cohort, the association between gene expression risk scores and two prognostic indicators was corroborated and visualized through heatmaps (Figure 3E). Furthermore, both univariate and multivariate analyses confirmed that metastasis and the risk score are independent prognostic factors for osteosarcoma (Figure 3F,G).

### 3.3 Validation of risk models

**FIGURE 4:**
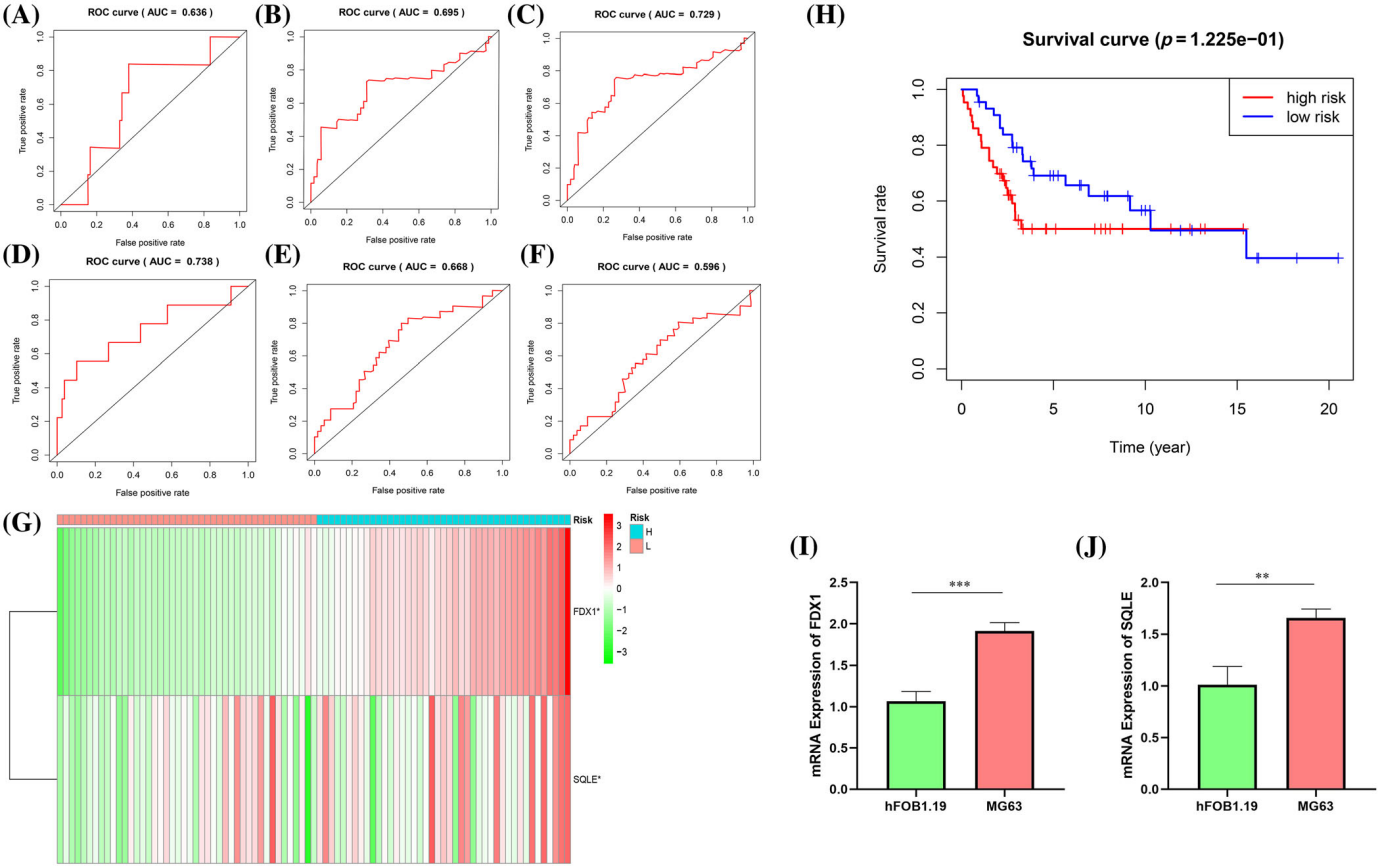
Validation of model for predicting the prognosis of osteosarcoma (OS) patients. (A, B, C) The training set of the receiver operating characteristic (ROC) curve for evaluating the prediction efficiency of the prognostic signature. (D, E, F) The testing set of the ROC curve for evaluating the prediction efficiency of the prognostic signature. (G) Expression patterns of two selected prognostic genes in high‐and low‐risk groups for testing set. (H) The Kaplan–Meier analysis showed that patients in the low‐risk group presented better OS than those in the high‐risk group for testing set. (I) mRNA expression of FDX1. (J) mRNA expression of SQLE.

The prognostic risk models were assessed using the area under the curve (AUC) of the ROC curves at 1‐, 3‐, and 5‐year intervals, as derived from the training set data. The resultant AUC values were 0.636, 0.695, and 0.729, respectively (Figure 4A–C). Similarly, the analysis of the validation set yielded AUC values of 0.738 for 1‐year, 0.668 for 3‐year, and 0.596 for 5‐year ROC curves (Figure 4D–F). The expression profiles of FDX1 and SQLE in the two identified risk groups corresponded with the patterns observed in the training set (Figure 4G). Importantly, survival rates in the low‐risk group of the validation set consistently exceeded those in the high‐risk group (Figure 4H). The hFOB 1.19 and MG‐63 cells were utilized to validate the mRNA expression of SQLE and FDX1 in osteosarcoma (OS). The mRNA levels of SQLE and FDX1 in these OS cell lines were elevated in the hFOB 1.19 cell line (Figure 4I,J), aligning with our previous observations.

### 3.4 Construction and verification of a nomogram model

**FIGURE 5:**
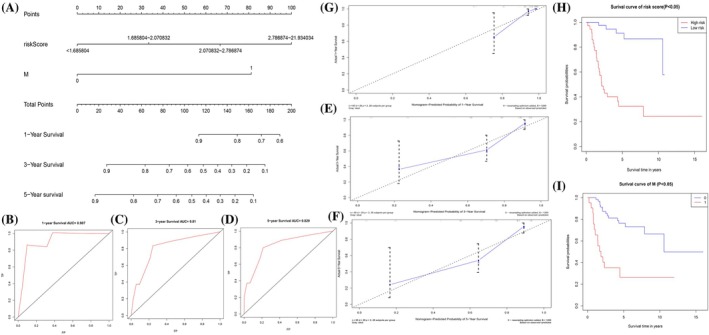
Construction and validation of a nomogram for predicting the prognosis of osteosarcoma (OS) patients. (A) Nomogram for predicting the 1‐, 3‐, and 5‐years OS of osteosarcoma patients in the TARGET‐OS cohort. (B, C, D) The receiver operating characteristic (ROC) curves of the nomograms compared for 1‐, 3‐, and 5‐years OS in osteosarcoma patients, respectively. (E, F, G) Calibration curves for validating the established nomogram. (H) Kaplan–Meier survival curves stratified according to risk scores. (I) Kaplan–Meier survival curves stratified according to metastasis.

We developed a nomogram utilizing independent prognostic factors for predicting overall survival (OS) at 1‐, 3‐, and 5‐year intervals, as shown in Figure 5A. The model's predictive performance was evaluated using the concordance index (c‐index) and calibration curves (Table 1). The ROC curves depicted AUC values of 0.907, 0.810, and 0.829 for 1‐, 3‐, and 5‐year overall survival correspondingly, as illustrated in Figure 5B–D, indicating high predictive accuracy. Calibration plots further confirmed the model's reliability (Figure 5E–G). Survival analysis demonstrated a statistically significant difference in early versus late survival rates (*p* < .001). Additionally, the survival analysis indicated that patients in the low‐risk cohort experienced significantly better overall survival than those in the high‐risk cohort (*p* < .05; Figure 5H,I).

**TABLE 1:** C‐index for verifying the validity of Norman diagram.

C‐index	Dxy	S.D.	*n*
0.8064126	1.6128252	0.9303953	−84.0000000
Uncensored	Relevant pairs	Concordant	Uncertain
−28.0000000	−3305.0000000	−639.0000000	−3833.0000000

### 3.5 Expression pattern of immune cell infiltration based on risk model

**FIGURE 6:**
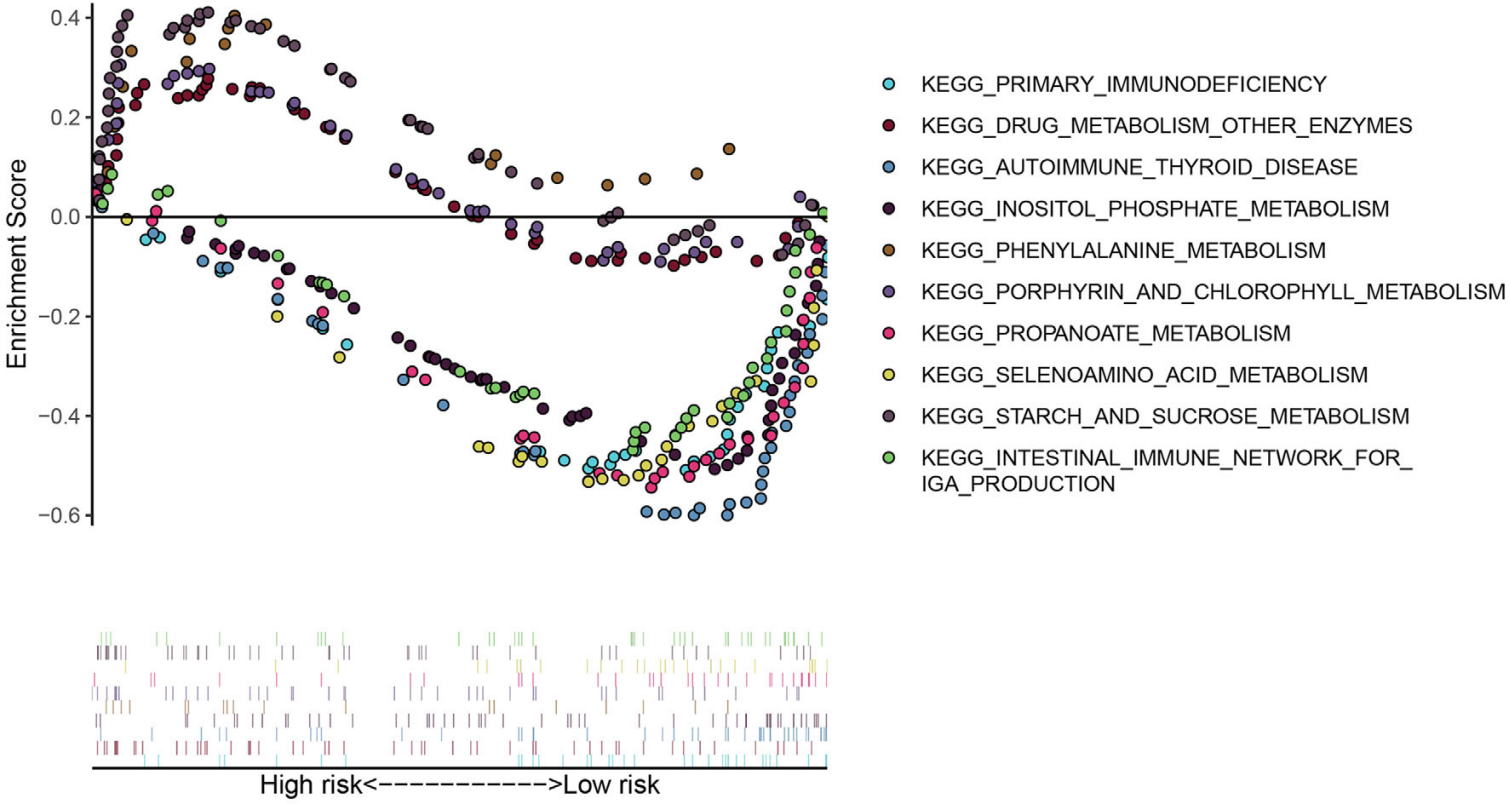
Gene set enrichment analysis analysis between high‐risk and low‐risk groups.

**FIGURE 7:**
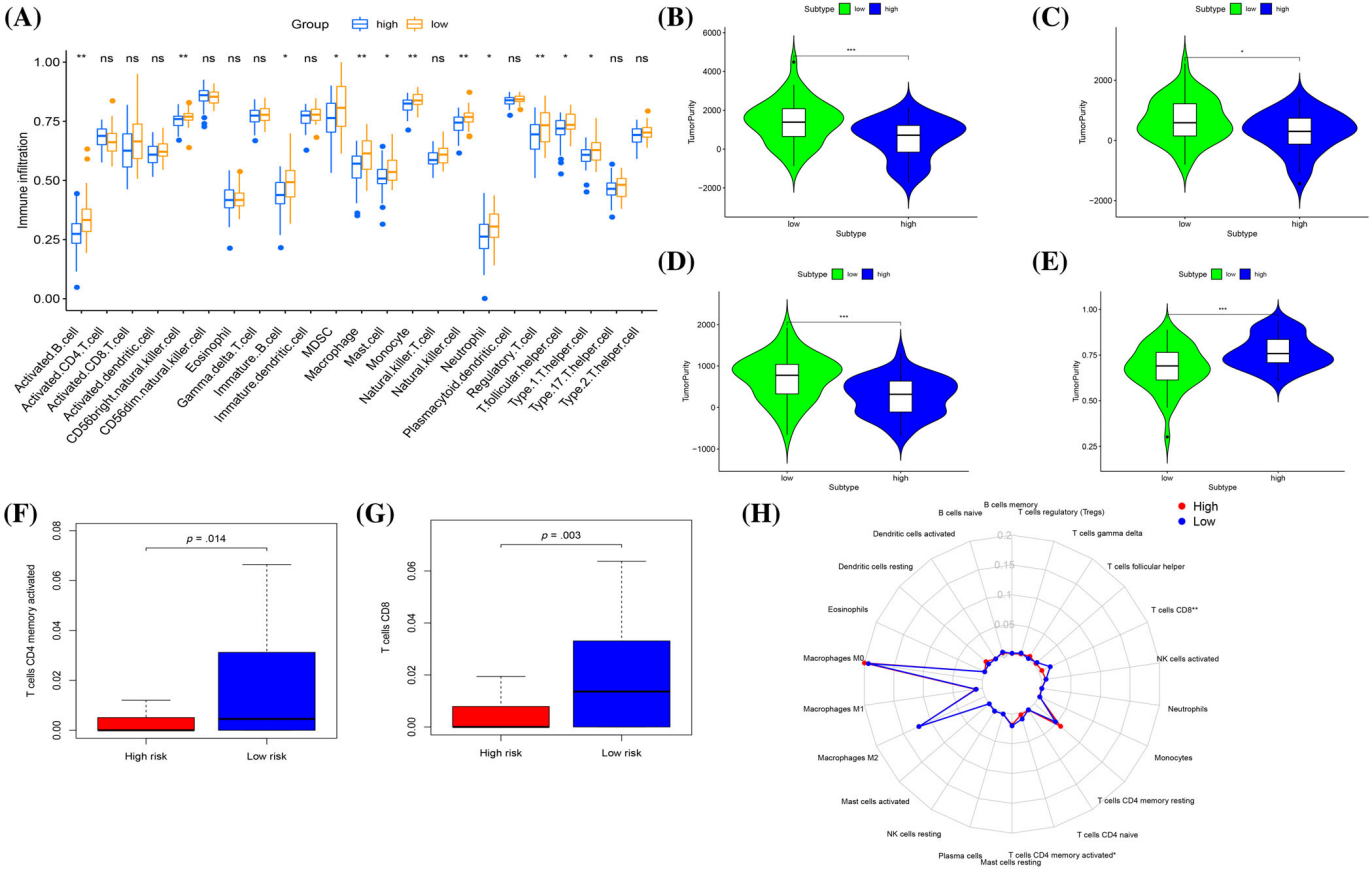
Prognosis and TME characteristics in two clusters for osteosarcoma (OS) patients. (A) Box plot for the TME cells in distinct risk groups derived from OS patients based on the single‐sample gene set enrichment analysis. The asterisks represented the statistical *p* value (*p < .05; **p < .01; ***p < .001). (B, C, D, E) Immune, stromal, ESTIMATE and TumorPurity scores within the low‐ and high‐risk groups. (F, G) Expression of two key immune cells in two groups. (H) Summary of the immune cells' abundance for different risk groups.

To clarify the role of the proposed model, we conducted GSEA to explore its association with potential signaling pathways. The results demonstrated connections between the risk model and various immune pathways, notably PRIMARY IMMUNODEFICIENCY, as depicted in Figure 6. We further assessed the relationship between immune cell infiltration and risk score through ssGSEA, shown in Figure 7A. The high‐risk group exhibited marked reductions in expression levels across multiple immune cells, including activated B cells, CD56bright natural killer cells, immature B cells, myeloid‐derived suppressor cells, macrophages, mast cells, monocytes, natural killer cells, neutrophils, regulatory T cells, T follicular helper cells, and type 1 T helper cells, with statistical significance indicated by *p* < .05; *p* < .01; and **p < .001. These findings suggest the substantial influence of immune cell concentration and infiltration on osteosarcoma prognosis. Comparative analysis using the ESTIMATE algorithm indicated that the high‐risk group possessed lower total, immune, and stromal scores, yet exhibited increased tumor purity, as shown in Figure 7B–E. Additionally, CIBERSORT algorithm analysis highlighted significant disparities in immune cell composition between the higher and lower groups, with a pronounced depletion in CD4 memory activated T cells and CD8 T cells in the high‐risk group, as detailed in Figure 7F–H.

### 3.6 Immune checkpoints and immune evasion related to risk models

**FIGURE 8:**
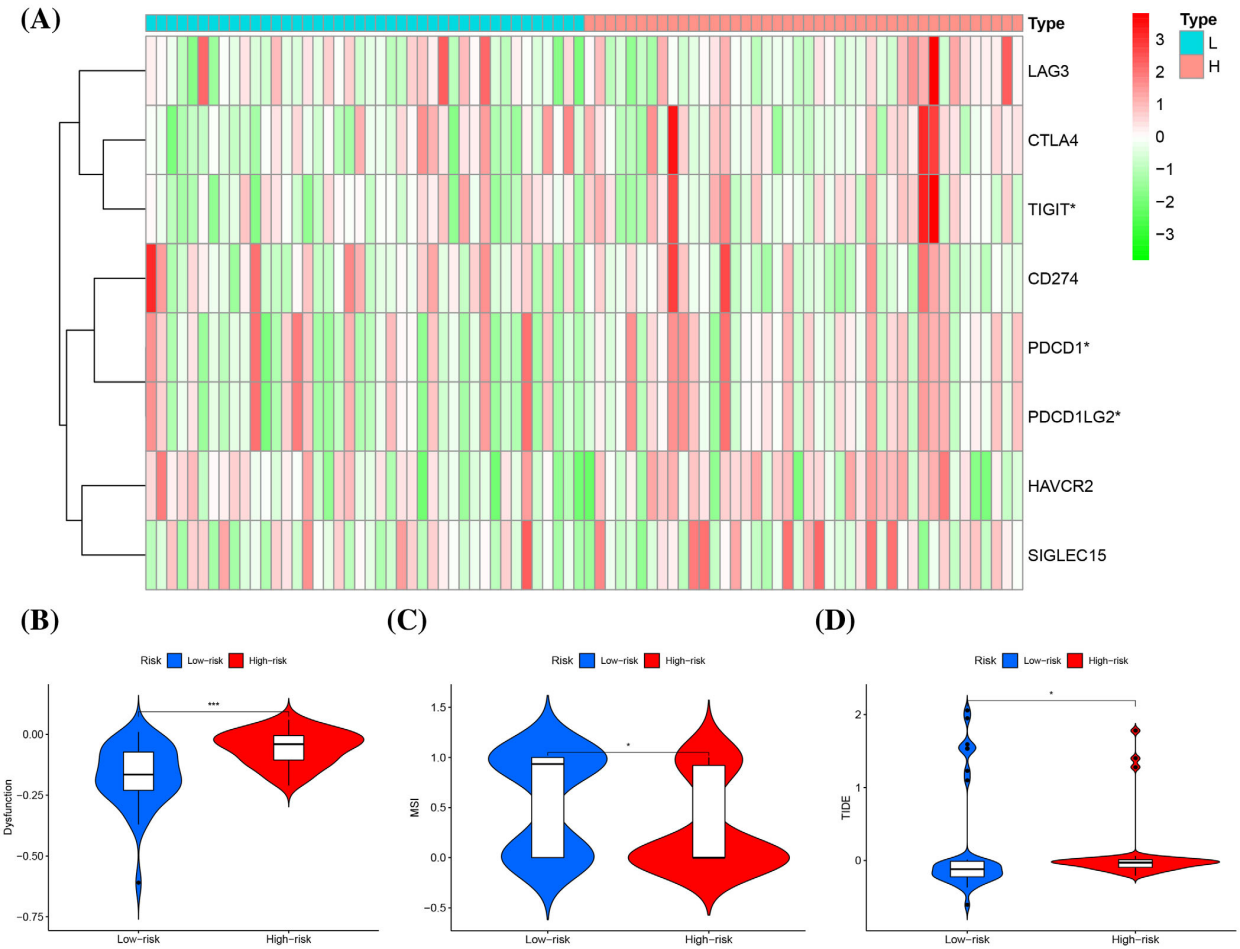
Sensitivity of immunotherapy and immune escape in high and low wind groups. (A) Differences between the two groups of eight immune checkpoints. (B, C, D) Comparison of tumor immune dysfunction and exclusion in two risk groups.

The study evaluated the expression patterns of various genes related to immune checkpoints. SIGLEC15, TIGIT, CD274, HAVCR2, PDCD1, CTLA4, LAG3, and PDCD1LG2 are involved. Significant differences were observed in the expression levels of TIGIT, PDCD1, and PDCD1LG2 between the two risk groups. The lower group demonstrated elevated expression of these immune checkpoints, as depicted in the heatmap (Figure 8A). In stark contrast, the high‐risk group showed increased TIDE scores and dysfunction scores, suggestive of enhanced immune escape and regulatory imbalances. Furthermore, the high‐risk group presented with a lower MSI score relative to the low‐risk group, implying a reduced presence of immune cells with antitumorigenic activities in patients with higher risk (Figure 8B–D). These results underscore the promising therapeutic potential of immune checkpoint inhibitors in cancer management.

### 3.7 Potential drug screening based on risk models

**FIGURE 9:**
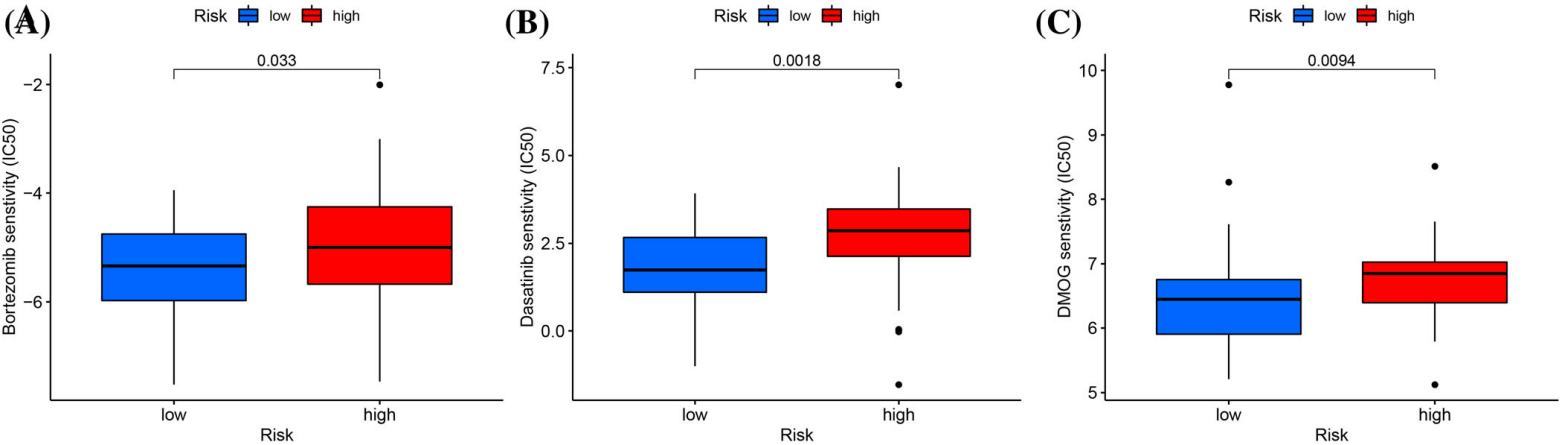
Predicting the responsiveness of osteosarcoma to chemotherapy based on risk model. (A) Bortezomib sensitivity. (B) Dasatinib sensitivity. (C) DMOG sensitivity.

The study identifies three drugs—bortezomib, dasatinib, and DMOG—as pertinent to osteosarcoma treatment based on the risk model depicted in Figure 9A–C. Contrary to the higher cohort, the lower group demonstrated increased sensitivity to these agents. These results extend the pharmacological repertoire for osteosarcoma management; however, additional research is required to fully understand their mechanisms of action. The statistical significance underpinning the drug sensitivity analysis is affirmed with a *p*‐value less than .05.

## 4 DISCUSSION

The malignancy ranks of osteosarcoma as the most commonly occurring tumor originating in bone tissue, in adolescents and young adults, noted for its pronounced invasive and metastatic capabilities.^20^ Current treatment modalities for osteosarcoma patients encompass surgery, radiotherapy, chemotherapy, and neoadjuvant chemotherapy.^21^ Despite these interventions, the overall survival rates are discouraging, especially in advanced disease stages, owing to osteosarcoma's aggressive nature.^22^, ^23^ Furthermore, resistance to conventional chemotherapy compounds the clinical challenge,^23^ necessitating alternative treatment approaches, such as promoters of tumor cell apoptosis, antiangiogenesis drugs, and immunotherapy.^24^, ^25^ Nevertheless, the clinical efficacy and the underlying mechanisms of these emerging therapies remain to be fully elucidated. A thorough understanding of osteosarcoma's molecular pathology is imperative for the identification of critical biomarkers for early diagnosis, targeted treatment, and prognosis.

Recent interest in the cellular death pathways of ferroptosis and cuproptosis has surged within tumor research. Since ferroptosis's introduction in 2012, studies have extensively explored its pathogenesis and therapeutic implications, affirming the link between intracellular reactive oxygen species levels and tumor biology.^26^, ^27^ Research suggests that ferroptosis‐inducing drugs hold promise in counteracting chemotherapy resistance associated with apoptosis.^28^, ^29^ Likewise, mounting evidence indicates the significance of copper‐induced cytotoxicity within tumor growth, immunity, and therapy.^4^ Despite numerous studies, the combined genetic activity of ferroptosis and cuproptosis in tumors remains enigmatic, underscoring the need for in‐depth research into these cellular death pathways to inform novel antitumor strategies.

In our study, we scrutinized the influence of 71 coexpressed genes on patient survival, identifying 34 regulatory genes significantly impacting prognosis. Higher gene expression levels were associated with better outcomes, exemplified by genes such as ACSF2, ACSL3, and G6PD. Conversely, genes like AIFM2 and AKR1C2 with lower expression correlated with poorer prognosis. Univariate Cox and LASSO regression analyses pinpointed two prognostic genes: FDX1 and SQLE. Immune profiling disclosed variations in immune cell populations between risk groups, with GSEA highlighting the primary immunodeficiency pathway in the low‐risk category. Notably, CD8^+^ T cell metabolism's role in anticancer immunity emerged as a critical factor.^30^ Further exploration of tumor‐associated immune checkpoints and the risk model is underway, revealing the high expression of TIGIT, PDCD1, and PDCD1LG2 in the low‐risk group. Our findings suggest that immunotherapy, indicated by TIDE, Dysfunction, and MSI scores, may yield greater efficacy in these patients. Additionally, chemotherapy sensitivity, represented by IC50 values for drugs such as Bortezomib and Dasatinib, underscores potential treatment avenues. Despite intriguing results, this study's limitations include an incomplete understanding of ferroptosis and cuproptosis in osteosarcoma and a need for larger datasets to minimize sampling bias. Future research will delve further into these cellular death mechanisms and validate findings with more clinical samples.

## AUTHOR CONTRIBUTIONS

Yingnan Ji: Writing—original draft; experiments parts. Zhenhua Ji: Formal analysis; conceptualization. Yudong Ma: Methodology and software. Lv Liu: Deal with revision process. Yu Liu: Validation; visualization. Xiaodan Wu: Resources; data curation; writing—review and editing. Qi Zhu: funding acquisition; project administration; and supervision.

## CONFLICT OF INTEREST STATEMENT

The authors declare that they have no competing interests.

## Data Availability

The original contributions presented in the study are included in the article/Supplementary Material, further inquiries can be directed to the corresponding author.
